# Clozapine administration enhanced functional recovery after cuprizone demyelination

**DOI:** 10.1371/journal.pone.0216113

**Published:** 2019-05-09

**Authors:** Nikki Templeton, Bronwyn Kivell, Amy McCaughey-Chapman, Bronwen Connor, Anne Camille La Flamme

**Affiliations:** 1 Centre for Biodiscovery, School of Biological Sciences and School of Chemical and Physical Sciences, Victoria University of Wellington, Wellington, New Zealand; 2 Department of Pharmacology and Clinical Pharmacology, Centre for Brain Research, School of Medical Sciences, FMHS, the University of Auckland, Auckland, New Zealand; 3 Malaghan Institute for Medical Research, Wellington, New Zealand; Instituto Cajal-CSIC, SPAIN

## Abstract

The atypical antipsychotic agent, clozapine, is used to treat a variety of neurological disorders including schizophrenia and Parkinson’s disease and readily crosses the blood brain barrier to interact with a wide range of neuroreceptors including those for dopamine and serotonin. Recent work has shown that clozapine can reduce neuroinflammation in experimental autoimmune encephalomyelitis, a neuroinflammatory model of multiple sclerosis (MS) and mediates its effects in the central nervous system. To further characterise the protection provided by clozapine, the cuprizone model of demyelination was used to assess the effect of clozapine treatment on the cellular events surrounding demyelination and remyelination. Using this model of non-immune demyelination, we found that clozapine administration was unable to prevent demyelination, but when administered post demyelination, was able to enhance the rate of functional recovery. The more rapid improvement of clozapine-treated mice correlated with a decreased level of astrocyte and microglial activation but only modestly enhanced remyelination. Together, these studies highlight the potential of clozapine to support enhanced functional recovery after demyelination, such as that occurring during MS.

## Introduction

Multiple Sclerosis (MS) is a disease of the central nervous system (CNS) affecting approximately 2.3 million people worldwide [1]. Disease pathology is characterised by demyelinating lesions forming in the CNS and an infiltration of self-reactive lymphocytes [2]. Current therapies for MS consist primarily of immune-modifying treatments designed to slow disease progression and decrease relapse frequency [3]. The current MS therapeutics available are limited in their ability to treat progressive forms of MS; therefore, alternative MS therapies are still required that can reduce disability in progressive disease [4].

Atypical antipsychotics, including clozapine, are used to treat a variety of neurological disorders such as schizophrenia, autism, and Parkinson’s disease, and these medicines readily cross the blood brain barrier where they are able to interact with a wide range of neuroreceptors including those for dopamine and serotonin [5]. Recent work has also shown that atypical antipsychotics can reduce neuroinflammation in schizophrenic individuals [6–8], and that orally administered clozapine can reduce disease in experimental autoimmune encephalomyelitis (EAE) [9, 10]. To further characterise the protection provided by clozapine, the cuprizone model of demyelination was used to assess the effect of clozapine treatment on cellular events surrounding demyelination and remyelination. During the cuprizone model the blood brain barrier remains intact, so the direct effect of clozapine in the CNS can be assessed without the accompanying immune infiltration seen in the EAE model, leading to lesions, which lack peripheral immune infiltrate and can be found in progressive disease [4, 11, 12]. In addition to investigating the effects on demyelination and remyelination, behavioural assays were carried out to assess potential functional improvements in the motor deficits often seen in the cuprizone model. Together, this study aimed to assess the therapeutic potential of clozapine in treating the demyelination that characterizes progressive MS.

## Materials and methods

### Animals

Female C57BL/6 mice were purchased from the Malaghan institute of Medical Research (Wellington, NZ) and were used between 6–8 weeks of age. All experimental procedures were approved by the VUW Animal Ethics Committee (2013-R18). Animals were housed with a 12-hour light/dark cycle, with behavioural assays carried out during the dark cycle. No special housing conditions or anaesthetics were required, as cuprizone and clozapine were administered in the food and drinking water, respectively. All efforts were made to minimize animal suffering, and no animals reached a humane endpoint (i.e. loss > 20% of body weight *and* signs of suffering such as lack of grooming, hunched posture, or reduced activity). The total number of animals used is indicated in the figure legends and includes all animals from 2–3 independent experiments as indicated in the figure legends.

### Administration of 0.3% cuprizone and 60 mg/kg clozapine

Food and water were available *ad libitum*. Pelleted mouse feed was ground before 0.3% cuprizone (Sigma, St. Louis, MO USA) was mixed through. Powdered feed was made freshly every 2–3 days and was replaced daily. Clozapine, kindly supplied by Douglas Pharmaceuticals Ltd (Auckland, NZ), was dissolved in 0.1 M acetic acid to a concentration of 6 mg/ml before being diluted in the drinking water to deliver a daily dose of 60 mg/kg based upon an average water consumption of 3 ml/day as previously reported [9].

### Rotarod behavioural assay

Mice were placed on the rotating rod (i.e. rotarod) at a fixed speed of 28 rpm. A timer was started as soon as the mouse’s tail was released and length of time the mouse was able to remain walking on the rotating rod was recorded. Maximum length of performance on this task was 120 seconds. If a mouse fell, or did 2 consecutive rotations the rod was stopped and the time recorded. A fall within the first 5 seconds was discounted as due to improper placement and a brief rest period was given before a retest.

### Beam test behavioural assay

Mice were placed in the middle of a horizontal beam suspended between two props, 45 cm above the bench. Time taken for mice to reach either end of the beam was recorded, with the best time of two repeats being used for analysis. Maximal test length was 30 seconds, after which the animal was assigned the maximum score of 30 if it failed to complete the task.

### Tissue processing

Animals were transcardially perfused with PBS followed by 4% paraformaldehyde (PFA) (Acros Organics, Geel, Belgium) in 1 X PBS. Brains were dissected out and placed in 4% PFA overnight before being transferred to 70% ethanol. Two mm segments containing the region of interest were processed in a tissue processor (Leica TP1020, Wetzler, Germany) and embedded using a Leica embedding station (EG-1160). Sections of 5–7 μm thickness were used for histological staining.

### Histological techniques

Myelin was detected using luxol fast blue stain (LFB; Sigma) followed by cresyl violet counterstain (Sigma) to identify neuronal tissue structure. The guideline for scoring is shown in S1 Fig. Astrocytes were detected using anti-glial fibrillary acid protein (GFAP, 1:1000 DAKO, Glostrup, Denmark) overnight at 4°C followed by donkey anti-rabbit IgG at 1:500 (Jackson Immunochemicals, PA, USA) for 3 hours. Slides were then stained with an ABC staining kit (Vector Labs, CA, USA) followed by Vector VIP substrate (Vector) according to the manufacturer’s instructions. Microglia were detected using ionized calcium binding adaptor molecule 1 (Iba1, 1:250 Abcam, Cambridge, UK) and myelin was detected using myelin basic protein (MBP, 1:500 Millipore, Burlington, MA, USA). Both antibodies were incubated on sections overnight at 4°C followed by donkey anti-goat IgG and donkey anti-rat IgG at 1:500 (Sigma) for 3 hours. Sections were then stained with extravidin peroxidase (1:500, Sigma) for 1 hour and developed with diaminobenzidine/nickel sulfate (DAB/Ni, Sigma) for up to 20 minutes. Sections of corpus callosum were assessed using a photomicroscope with an Olympus DP72 camera (Tokyo, Japan) and CellSens software (version 1.16; Olympus, Tokyo, Japan) at 20 x magnification.

### Quantification of LFB, MBP, Iba-1, and GFAP tissue staining

All tissue staining was assessed by two different methods: quantification by ImageJ and categorical scoring by blinded assessors. For ImageJ quantification, images of corpus callosum sections were converted to an RGB image stack and the colour inverted to assess specific staining intensity. For LFB and GFAP, the full corpus collosum image was quantified while for MBP and Iba-1, a grid was applied and randomly selected squares within the corpus collosum were quantified for intensity and the ventricle used as a background control. For blinded assessor quantification, images of corpus callosum sections were presented in a random order to 3–4 observers blinded to treatments and scored on a scale from 0–3 with 0 being low expression and 3 being strong expression as previously used in histological studies [13]. The guideline for scoring is shown in S1 Fig. The average score for each treatment group was then calculated.

### Statistical analyses

All data were graphed and analysed using GraphPad Prism version 7 (La Jolla, CA, USA). For experiments with >2 groups, one or two-way ANOVA was used followed by the recommended multiple comparisons test as indicated in figure legends.

## Results

### Clozapine administration did not alter disease parameters during chronic cuprizone intoxication

**Fig 1:**
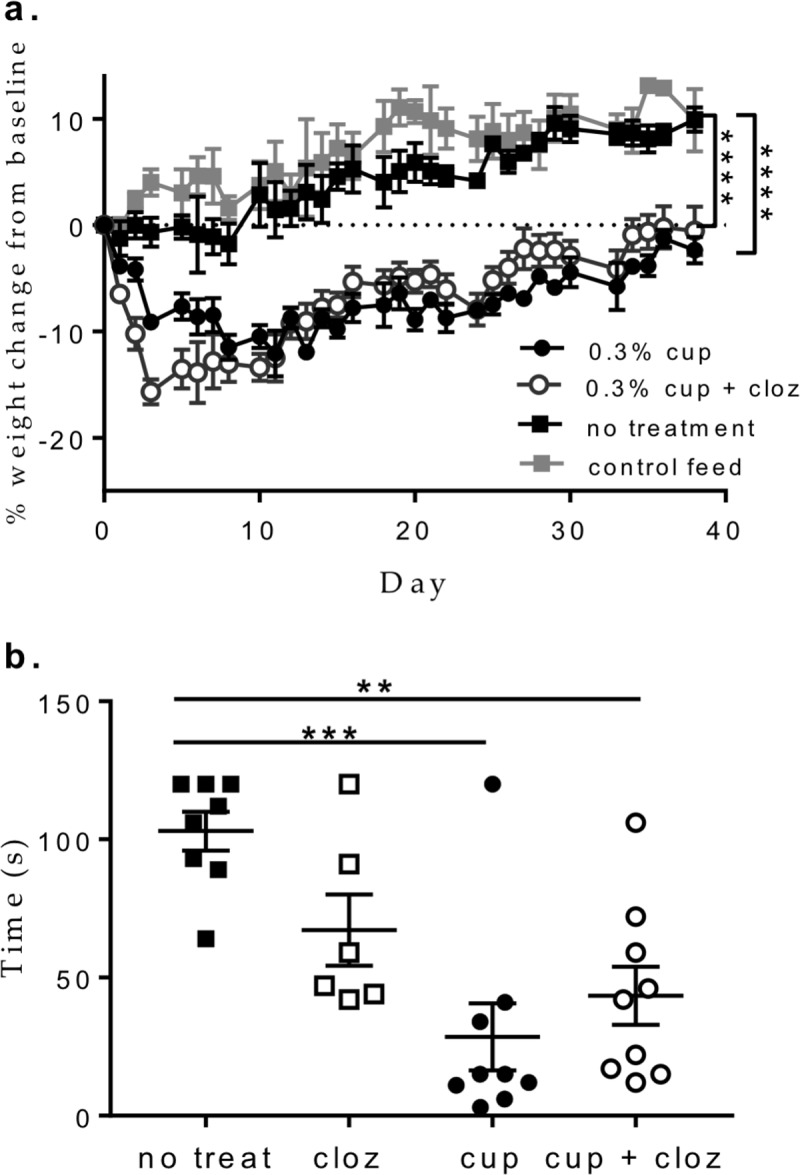
Clozapine did not prevent weight loss or functional deficits induced by chronic cuprizone intoxication. a. 6–8 week old female C57Bl/6 mice were fed a diet containing 0.3% cuprizone mixed into ground mouse feed. Food was replaced daily and water was available ad libitum. Clozapine (60 mg/kg/day) was administered orally in the drinking water, and animals were weighed daily. Shown are the means ± SEM of individual mice (n = 10/group for cuprizone +/- clozapine, n = 5 clozapine alone, and n = 4 control feed). Two-way ANOVA with Tukey’s multiple comparison test ****p<0.0001 compared to control feed. b. On the final day of cuprizone feeding, mice were placed on a rotating rod at a fixed speed of 28 rpm, and the time mice were able to continue walking was recorded. Maximum test duration was 120 seconds with the better of 2 attempts per animal recorded. Shown are the means and SEM of individual mice (n = 9/group for cuprizone +/- clozapine, n = 6 clozapine alone, and n = 8 no treatment) from 2 independent experiments. One-way ANOVA with Holm-Sidak’s multiple comparison test (**p<0.01, ***p<0.001).

To understand if clozapine were able to alter cuprizone-mediated demyelination, mice were fed a 0.3% cuprizone diet for 6 weeks and administered clozapine (60 mg/kg/day) or its vehicle (0.1 M acetic acid) in drinking water. No sign of stress (e.g. lack of grooming behaviour) other than weight loss was observed in all groups fed cuprizone compared to untreated mice or mice treated with clozapine alone (Fig 1A). Mice receiving cuprizone rapidly lost weight over the first 10 days before weight stabilised. Treatment with clozapine did not prevent the cuprizone-mediated weight loss (Fig 1A).

After 6 weeks on a cuprizone diet, the rotarod was used to assess motor coordination. As expected, feeding with 0.3% cuprizone alone reduced the length of time animals were able to remain on the rotarod at a fixed speed of 28 rpm (Fig 1B) and similar results were found with mice treated with 0.3% cuprizone and the clozapine vehicle (S2A and S2B Fig). Mice receiving 0.3% cuprizone had a significantly decreased performance compared to the no cuprizone control group, but this decreased performance was not improved significantly by clozapine (Fig 1B). Although the average performance for animals in the clozapine-treated, cuprizone group was 43.4 ± 10.5 seconds compared with 28.3 ± 12.7 seconds in the untreated, cuprizone group, this difference did not reach significance (Fig 1B).

### Clozapine administration did not reduce demyelination or astrocyte activation during chronic cuprizone intoxication

**Fig 2:**
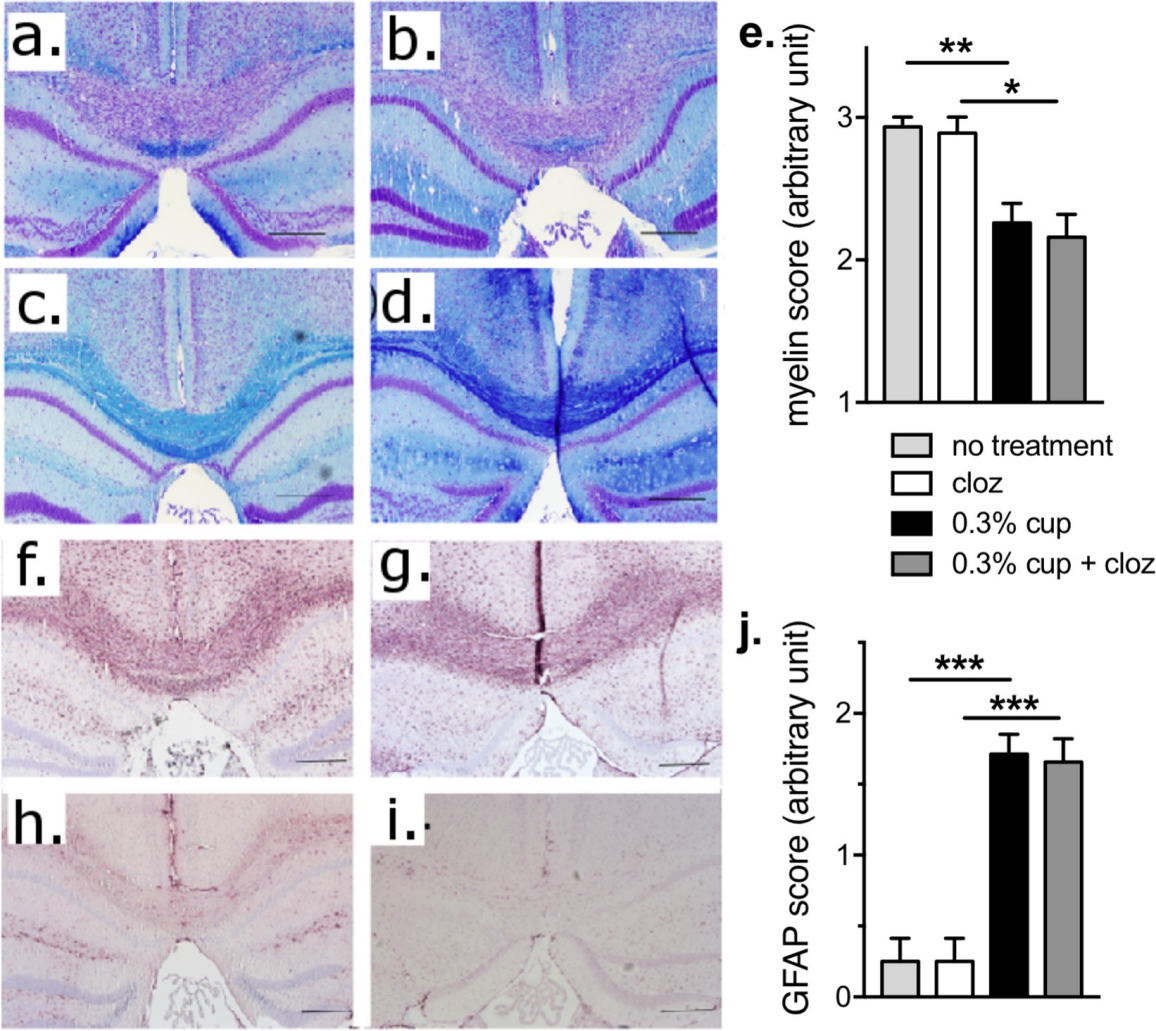
Clozapine did not prevent demyelination or alter astrocyte activation induced by chronic cuprizone intoxication. 5–7 μm sections were stained with LFB to detect myelin and cresyl violet counterstain (a-d) with representative images showing (a,f) 0.3% cuprizone, (b,g) 0.3% cuprizone + 60 mg/kg clozapine (c, h) clozapine and (d,i) control. Consecutive sections were immunolabelled for GFAP (e-g). Scale bar = 100 μm. Sections were scored by individuals blinded to treatment groups to povide overal scores for demyelination (e) and astrocyte activation (j). (e & j) Shown are the means and SEM of individual animals (n = 8–10) from 2 independent experiments. Kruskal-Wallis test with Dunn’s multiple comparison (*p<0.05, **p<0.01, ***p<0.001).

To assess the effect of clozapine administration on demyelination, sections were stained with LFB and counterstained with cresyl violet to show cell nuclei. The area of interest was the corpus callosum, as this area is reproducibly targeted by cuprizone administration [14]. In all groups receiving cuprizone, demyelination was evident and was significantly increased compared to the control animals not receiving cuprizone (Fig 2A–2D). Moreover, clozapine treatment did not prevent or reduce demyelination in this model (Fig 2E).

To determine if clozapine affected astrocytes in this model, consecutive sections were assessed for astrocyte activity by immunolabeling for GFAP (Fig 2F–2I). As previously reported [15, 16], exposure to cuprizone in the diet led to mass activation of astrocytes, but treatment of cuprizone-fed animals with clozapine did not significantly reduce the level of astrogliosis (Fig 2J). Taken together these results show that clozapine treatment is unable to prevent cuprizone-induced demyelination or astrocyte activation during 6 weeks of 0.3% cuprizone administration.

### Clozapine enhanced functional recovery after cessation of cuprizone intoxication

**Fig 3:**
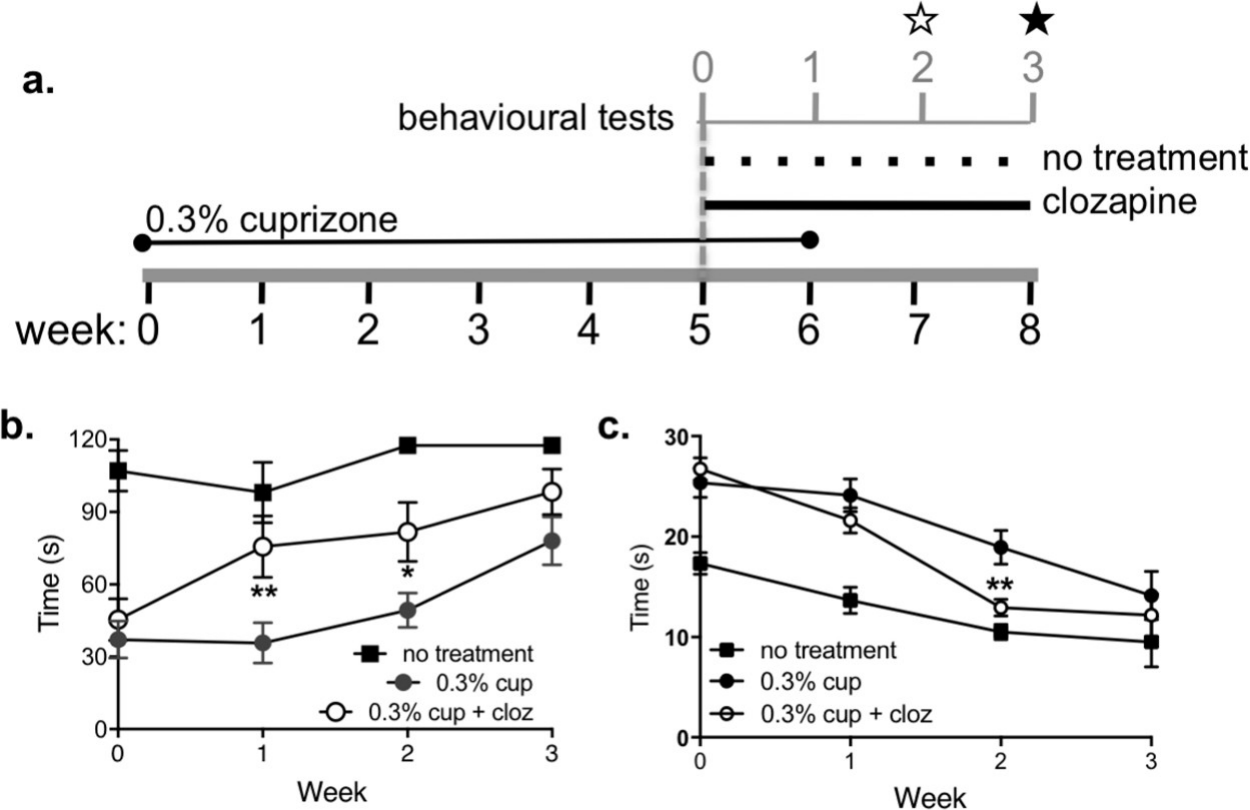
Clozapine enhanced recovery after cessation of cuprizone administration. (a) Experimental design for investigating remyelination events. Mice were fed a diet containing 0.3% cuprizone for 6 weeks. After the 5^th^ week of cuprizone, clozapine or vehicle treatment began. Cuprizone feeding continued until end of week 6 when mice were returned to a normal diet. Behavioural assays were carried out at indicted time points, b & c. Functional performance was assessed weekly using the rotarod at 28 rpm (b) and the beam test (c). Shown are means with SEM for 3 independent experiments with n = 6 (no treatment), 14 (0.3% cup), and 13 (0.3% cup + cloz). Two-way ANOVA with Holm-Sidak’s multiple comparison test (*p<0.05 and **p<0.01).

To assess the effect of clozapine treatment on remyelination, cuprizone was removed from the feed after 6 weeks and animals were monitored for another 2 weeks (Fig 3A). Treatment with clozapine began one week prior to withdrawal of cuprizone from the diet and behavioural assays were carried out weekly from the start of clozapine treatment (Fig 3A). As shown in Fig 3B, five weeks of cuprizone intoxication significantly reduced the performance of mice on the rotarod compared to healthy controls, and removal of cuprizone led to a progressive improvement over the recovery period. No difference was found in cuprizone-fed animals treated with clozapine vehicle or left untreated (S2 Fig); these groups were combined as a cuprizone control group. Treatment with clozapine led to significantly improved motor coordination, which was most evident after one week of clozapine treatment (Fig 3B). To measure grip strength and coordination, mice were also assessed on the beam test. Untreated, healthy mice showed improved performance over the testing period, and while cuprizone mice performed more poorly at each point, all mice receiving cuprizone also showed improvement over the recovery period. As with the rotarod test, clozapine treatment led to a significantly more rapid improvement in performance when compared to untreated cuprizone-fed animals (Fig 3C). Because the rotarod assesses gross motor function, it is more likely to detect early improvements following cuprizone withdrawal. In contrast, the beam test assesses fine motor coordination, which may require a longer period before improvements are evident. Overall, these results suggest that clozapine treatment promotes an earlier functional recovery after chronic demyelination.

### Clozapine administration led to reduced astrocyte and microglial activation one but not two weeks after cessation of cuprizone intoxication

**Fig 4:**
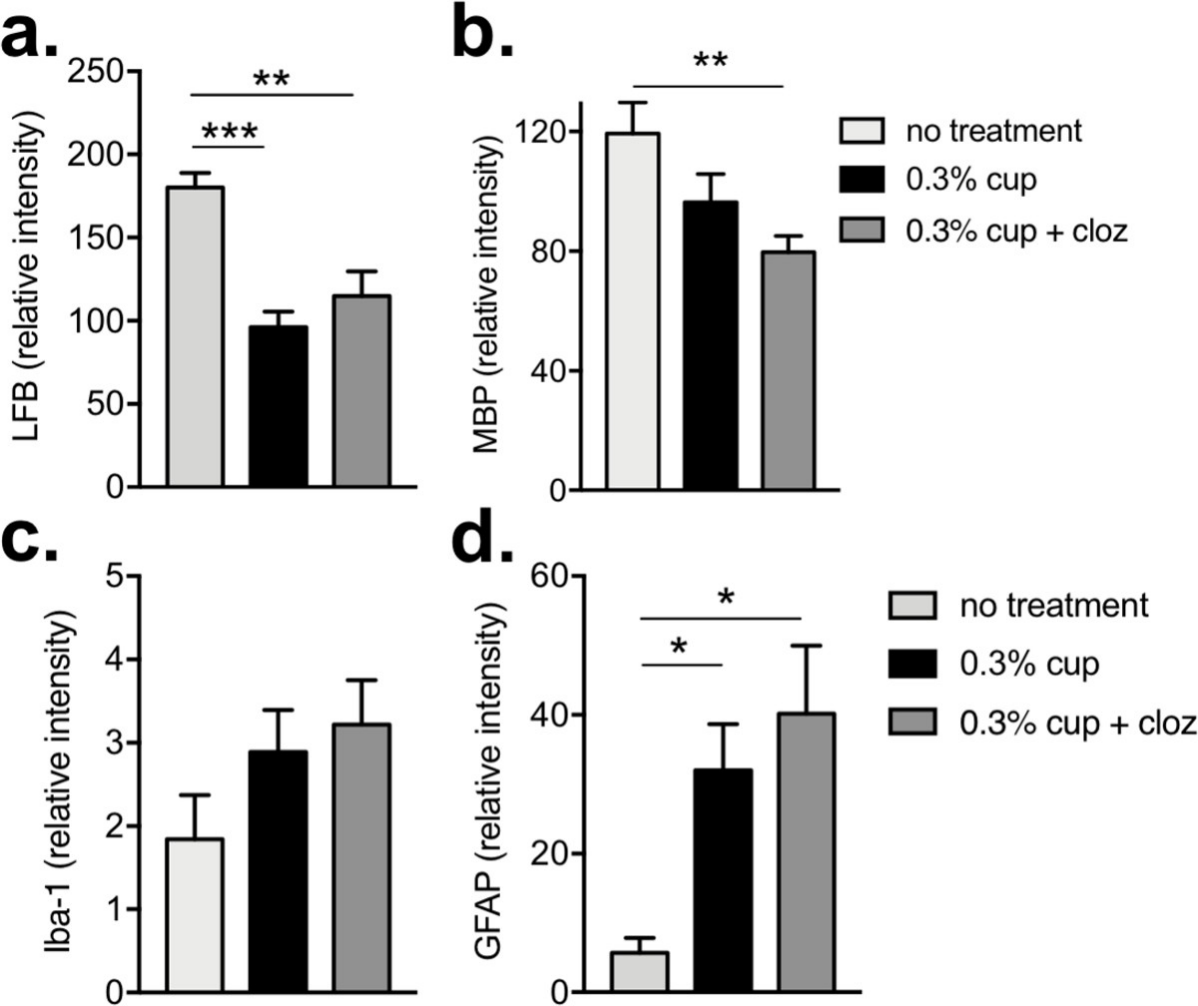
Clozapine did not significantly alter myelination or glial activation 2 weeks after cessation of cuprizone administration. Sections (timepoint noted by ★ in Fig 3A) were quantified using ImageJ to evaluate myelin by LFB (a) or MBP (b), Iba-1 (c) and GFAP (d). Shown are the means and SEM of individual mice with (a & d) n = 12 for cup and cup + cloz, n = 4 untreated and (b & c) n = 6 for cup, n = 9 cup + cloz, and n = 3 untreated from 3 independent experiments. One-way ANOVA with Holm-Sidak’s multiple comparison test (***p<0.001, **p<0.01, and *p<0.05).

To determine if the functional improvements correlated to enhanced myelination or reduced astrocyte and microglial activation, myelin, Iba-1, and GFAP levels were assessed two weeks after cuprizone removal (i.e. after 3 weeks of clozapine treatment; noted by ★ in Fig 3A). Interestingly, at this time point there was no significant difference in the level of myelin in the corpus callosum between the untreated, cuprizone-fed or the clozapine-treated, cuprizone-fed mice as assessed by LFB or MBP staining (Fig 4A and 4B). Similarly, clozapine-treated, cuprizone-fed animals also showed similar levels of microglial (Iba-1) and astrocyte (GFAP) activation to untreated, cuprizone-fed animals. This activation (i.e. Iba-1 and GFAP) was significantly increased compared to control animals (Fig 4C and 4D). These analyses were done using ImageJ quantification, but similar results were found using categorical scoring by blinded assessors as shown in Figs 2 and S3.

**Fig 5:**
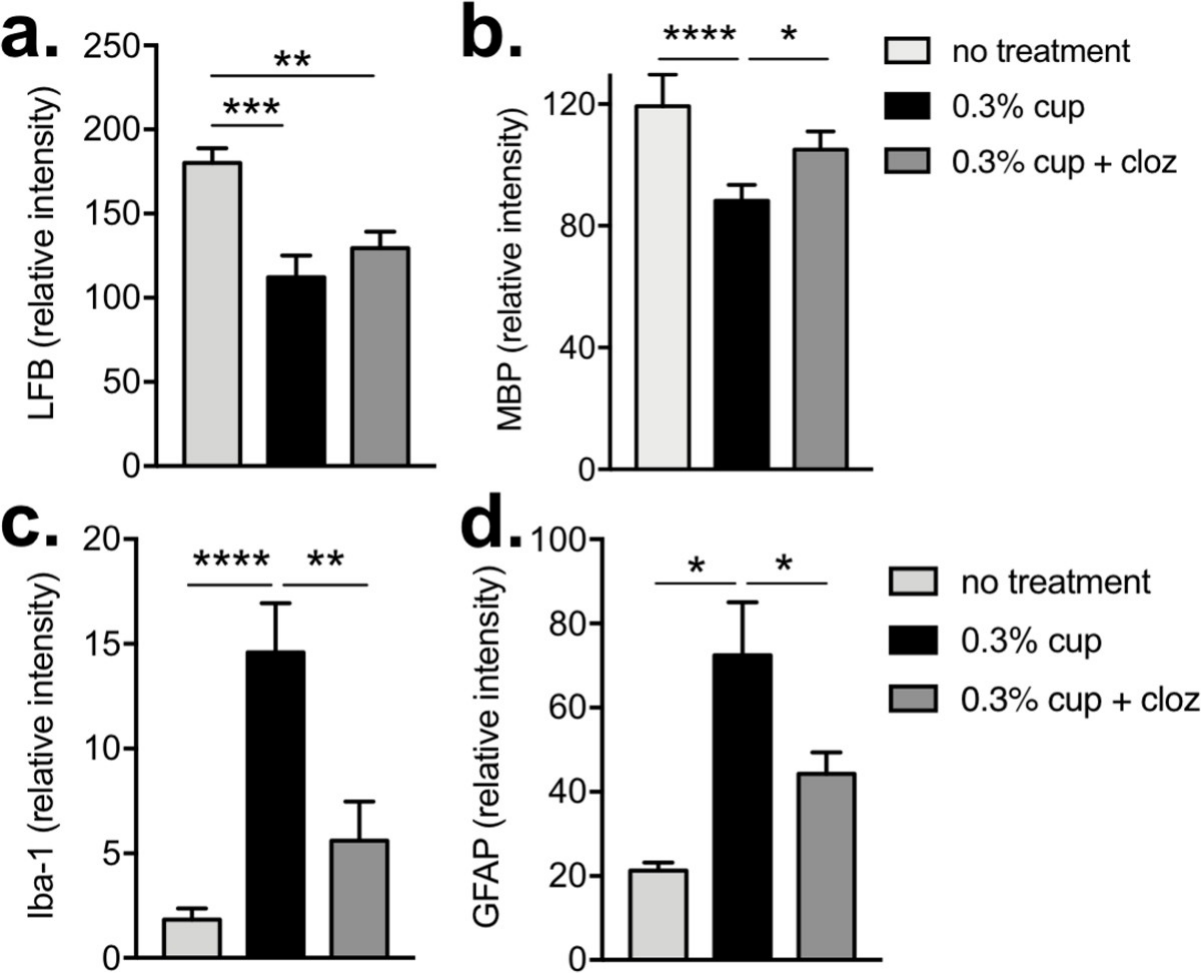
Enhanced clozapine-mediated functional recovery correlated to a modest increase in myelination and significant reduction in early glial activation. 6–8 week old female C57Bl/6 mice were fed 0.3% cuprizone mixed into ground mouse feed for 6 weeks and either untreated or treated with clozapine (60 mg/kg/day) after 5 weeks. After two (timepoint noted by ☆ in Fig 3A) weeks of clozapine treatment, mice were euthanized, the corpus callosum was stained for myelin by LFB (a) or MBP (b), Iba-1 (c) and GFAP (d), and sections quantified by ImageJ. Shown are the means and SEM of individual mice with (a & d) n = 12 for cup and cup + cloz, n = 4 untreated and (b & c) n = 7 for cup, n = 8 cup + cloz, and n = 3 untreated from 2 independent experiments. One-way ANOVA with Holm-Sidak’s multiple comparison test (****p<0.0001, ***p<0.001, **P,0.01, and *p<0.05).

Because behavioural improvements in the clozapine-treated mice were evident after 2 weeks of clozapine treatment, myelin and astrocyte levels were assessed one week after cessation of cuprizone (i.e. after 2 weeks of clozapine; noted by ☆ in Fig 3A). In agreement with the functional improvements, myelin levels at this earlier time point were modestly enhanced by clozapine treatment as assessed by MBP staining while the difference by LFB staining did not reach significance although it showed a similar trend (Figs 5A and 4B and S4). The greatest effect observed was the significant reduction in microglial and astrocyte activation in the corpus callosum in the clozapine-treated compared to untreated, cuprizone-fed animals supporting the idea that clozapine enhanced the early repair process post demyelination (Figs 5C and 4D and S4). Similar results were found using categorical scoring (S5 Fig). Finally, while cuprizone significantly reduced the number of mature oligodendrocytes (OGs) present in the corpus callosum (S6 Fig), treatment with clozapine did not alter the cuprizone-induced decrease in OGs at this time point when the reduction in astrocyte and microglial activation was most evident. Taken together, this work demonstrates that clozapine can promote early functional recovery and reduce microglial and astrocyte activation after wide scale, non-immune driven demyelination found in the cuprizone model.

## Discussion

The cuprizone model is a useful model to assess the effect of potential therapeutics, because both remyelination and demyelination occur at the same time, as happens in MS [11, 12]. To our knowledge the effect of *in vivo* clozapine treatment in the cuprizone model has not previously been investigated. Like previously reported results [17, 18], cuprizone treatment lead to a reduction in the mature OG population, with clozapine unable to protect this population (GST-pi transferase^+^ cells). Furthermore, a study looking at the role of quetiapine in remyelination after cuprizone-induced demyelination [19] showed that quetiapine treatment during the recovery period (after cuprizone was withdrawn) significantly improved behavioural performance and myelin restoration. Therefore, we hypothesised that like quetiapine, clozapine may have a protective effect in the cuprizone model and may be able to alter myelin levels.

While a modest enhancement of remyelination was detected in the corpus callosum following treatment with clozapine, the most significant effect observed by clozapine treatment was a significant increase in performance on the rotarod and a reduction in glial activation following 2 week of clozapine treatment and 1 week after cessation of the cuprizone diet. As the behavioural experiments progressed, all cuprizone-treated animals showed motor improvement from their starting point and this improvement in all cuprizone treated animals was correlated to reduced microglial and astrocyte activation and increased myelination in the corpus callosum. This repair is expected regardless of any therapeutic intervention, as once cuprizone is removed from the diet, remyelination has been shown to occur spontaneously [20, 21] and thus, clozapine appears to act as an enhancer of this repair process rather than acting on myelin directly.

We investigated whether clozapine would alter the astrocyte population such that their activation state would be reduced and could enable early remyelination by providing extra support to OGs or by altering the cytokine environment into a state more favourable to preventing damage. Astrocytes are the most abundant cell population in the CNS and have been implicated in many diseases, as well as playing a role in normal healthy brain function [22, 23]. Astrocytes are also responsible for the formation of glial scars around lesions, which limits damage to neighbouring tissue. Scar formation in MS, however, can also be damaging as it restricts the entry of OGs to produce myelin and can lead to chronic inflammation [24]. Our results indicate that clozapine reduces but does not completely abolish early astrocyte activation suggesting that it plays a regulatory not suppressive role on astrocyte functions.

Astrocytes have been shown to interact directly with OGs and also indirectly by acting on microglia and invading T cells. While invading T cells are not a substantial component of the cuprizone model, controlling the activation of microglia, potentially through the activation of astrocytes, could provide a promising therapeutic approach. As such, our findings that treatment with clozapine reduced not only astrocyte but also microglial activation early after a period of chronic demyelination support this strategy. This finding of reduced microglial activation is in agreement with previous studies reporting that clozapine can reduce the activation of microglia *in vitro* [25, 26] and *in vivo* [10]. However, because of the dual role reported for astrocytes and microglia in disease, it is difficult to determine if this decreased activation is protective or detrimental to the underlying neuronal structures [27] as the observed astrogliosis could be functioning to recruit microglia to clear debris and promote an environment conducive to repair, similar to the effect reported by Skripuletz et al [28]. The finding that the reduction in astrocyte activation correlated with reduced microglial activation; however, supports a positive role for the reduced glial activation. While further studies would be necessary to conclusively identify the impact of lowering astrocyte activation on recovery post demyelination, the concurrent improvement in functional performance in these mice is encouraging.

The improved performance seen in the rotarod assay could be a result of the reduced astrogliosis allowing for an environment more conducive to repair and remyelination. Previous studies have shown clozapine is able to reduce inflammation, with other studies showing that clozapine is able to reduce the cuprizone-induced decrease in maturation of NG2^+^ cells to O4^+^ cells and finally mature OGs capable of remyelinating axons [29]. It is possible that clozapine is enhancing the initial rate of remyelination following cuprizone withdrawal, and so by looking at earlier time points or by more sensitive methods than LFB or MBP staining, this effect may be more pronounced. The direct effect of clozapine on different OG populations *in vivo* is key to determining if this effect of clozapine on OGs is maintained, and could account for improved performance on behavioural tasks where MBP staining detected only a modest improvement in early levels of remyelination. Finally, it is also possible that the improved behavioural performance found in clozapine-treated animals could be due to a direct effect of the reduced inflammation and not related to changes in remyelination.

Previous studies have shown that clozapine and several other atypical anti-psychotic agents (e.g. risperidone and quetiapine) provide significant benefit in reducing disease in the EAE model of MS [9, 10, 30]. These studies reported significant effects when administered prophylactically as well as therapeutically with clozapine being the most effective compared to risperidone and quetiapine [9]. Although clozapine has been shown to reduce but not prevent Th1 and Th17 development and responses *in vitro* and *in vivo* and to enhance Treg differentiation, a direct effect on Th cells was not found to be responsible for clozapine’s protective effects in EAE [9, 10, 31]. Instead, the most significant effects were reported to be on CNS infiltration and innate cell activation in the CNS [9]. These findings are in agreement with this current study, which found that clozapine significantly reduced microglial and astrocyte activation in a model of demyelination that is independent of peripheral immune cells such as Th cells. Further research is needed to understand how clozapine directly affects inflammatory signalling in glial cells to regulate their activation.

In conclusion, this study found that clozapine administration enhanced the rate of recovery after cuprizone-driven demyelination. Given that the cuprizone-induced demyelination is believed to model the non-immune driven demyelination that occurs in some MS patients, this therapeutic effect highlights the potential for clozapine to aid in enhancing functional recovery during MS. Furthermore, because clozapine has been shown to have a strong protective effect in the EAE model [9], which models the neuroinflammatory processes in MS, together these studies indicate that clozapine is able to address both immune- and non-immune-mediated damage to the CNS in MS.

## Supporting information

S1 Fig Categorical scoring guide. Images were randomised, with images from all different treatment groups mixed together, and given to 3–4 blinded observers. Observers were asked to score the level of demyelination (LFB or MBP) based on 3- no visible demyelination, 2 = 0–30% demyelination, 1 = 30–60% demyelination and 0 = 60–90% demyelination. Astrocytes (GFAP) and microglia (Iba-1) were scored on a sliding scale with 3 indicating strong astrocyte activation through to 0 with no visible astrocyte activation.
(PDF)

S2 Fig Clozapine vehicle did not alter any disease parameters during or after cuprizone intoxication. Clozapine vehicle did not affect weight loss (a), rotarod performance (b), demyelination (c), or astrocyte activation (d) after 6 week treatment + 2 week recovery with clozapine vehicle and cuprizone compared to cuprizone alone. (a) n = 4/group; (b) no treatment (n = 6), cup alone (n = 7), and cup + veh (n = 6); (c) and (d) n = 2 per group, one experiment.
(PDF)

S3 Fig Clozapine did not significantly alter myelination or astrocyte activation 2 weeks after cessation of cuprizone administration. Sections (**★** in Fig 3A) were scored by individuals blinded to their treatment groups using a scale from 0 (low) to 3 (high) for myelin by LFP (a), MBP (b), Iba-1 (c), and GFAP (d). Shown are the means and SEM of individual mice with (a & d) n = 12 for cup and cup + cloz, n = 4 untreated and (b & c) n = 6 for cup, n = 9 cup + cloz, and n = 3 untreated from 3 independent experiments. Kruskal-Wallis test with Dunn’s multiple comparison test (****p<0.000, **p<0.01, and *p<0.05).
(PDF)

S4 Fig **Clozapine reduced microglial activation (a) and enhanced myelination (b) 1 week after cessation of cuprizone administration.** C57Bl/6 mice were fed 0.3% cuprizone diet for 6 weeks or a normal diet. Cuprizone-treated mice were treated with clozapine or vehicle beginning after 5 weeks of cuprizone intoxication. After two weeks of treatment (timepoint noted by ☆ in Fig 3A), mice were euthanized and 5–7 μm sections of corpus callosum were stained for Iba-1 (**a**) or MBP (**b**). Shown are representative images used to assess Iba-1 and MBP expression by ImageJ quantitation (Fig 5) or blinded observers (S5 Fig).
(PDF)

S5 Fig Clozapine enhanced myelination and reduced microglial and astrocyte activation 1 week after cessation of cuprizone administration. Sections (**★** in Fig 3A) were scored by individuals blinded to their treatment groups using a scale from 0 (low) to 3 (high) for myelin by LFP (a), MBP (b), Iba-1 (c), and GFAP (d). Shown are the means and SEM of individual mice with (a & d) n = 12 for cup and cup + cloz, n = 4 untreated and (b & c) n = 7 for cup, n = 8 cup + cloz, and n = 3 untreated from 2 independent experiments. Kruskal-Wallis test with Dunn’s multiple comparison test (****p<0.000, **p<0.01, and *p<0.05).
(PDF)

S6 Fig Following two weeks of clozapine treatment, clozapine did not significantly affect oligodendrocyte numbers. C57Bl/6 mice were fed 0.3% cuprizone diet for 6 weeks (+ cuprizone) or a normal diet (Cont.). Cuprizone-treated mice were treated with clozapine (Cloz) or untreated (UT) beginning week 5 of cuprizone intoxication. After two weeks of treatment mice were euthanized and 5–7 μm sections of corpus callosum were stained for mature oligodendrocytes using GST-pi transferase (indicated by arrows in a). The number of oligodendrocytes in the corpus callosum were counted by blinded observers (b). ***p<0.001 One-way ANOVA with Tukey’s post-test compared to all other groups (control n = 5, UT and Cloz-treated n = 4 per group).
(PDF)
