# Supplementary material for: Clozapine administration enhanced functional recovery after cuprizone demyelination
Source: PLoS One. 2019 May 9;14(5):e0216113. doi: 10.1371/journal.pone.0216113 (PMC6508663; doi:10.1371/journal.pone.0216113)
Supplement: S1 Fig — Images were randomised, with images from all different treatment groups mixed together, and given to 3–4 blinded observers. Observers were asked to score the level of demyelination (LFB or MBP) based on 3- no visible demyelination, 2 = 0–30% demyelination, 1 = 30–60% demyelination and 0 = 60–90% demyelination. Astrocytes (GFAP) and microglia (Iba-1) were scored on a sliding scale with 3 indicating strong astrocyte activation through to 0 with no visible astrocyte activation. (PDF) [file pone.0216113.s001.pdf]

Supplementary Figure 1: Categorical scoring guide. Images were randomised, with images from all different treatment groups mixed together, and given to 3-4 blinded observers. Observers were asked to score the level of demyelination (LFB or MBP) based on 3- no visible demyelination, 2= 0-30% demyelination, 1= 30-60% demyelination and 0= 60-90% demyelination. Astrocytes (GFAP) and microglia (Iba-1) were scored on a sliding scale with 3 indicating strong astrocyte activation through to 0 with no visible astrocyte activation.

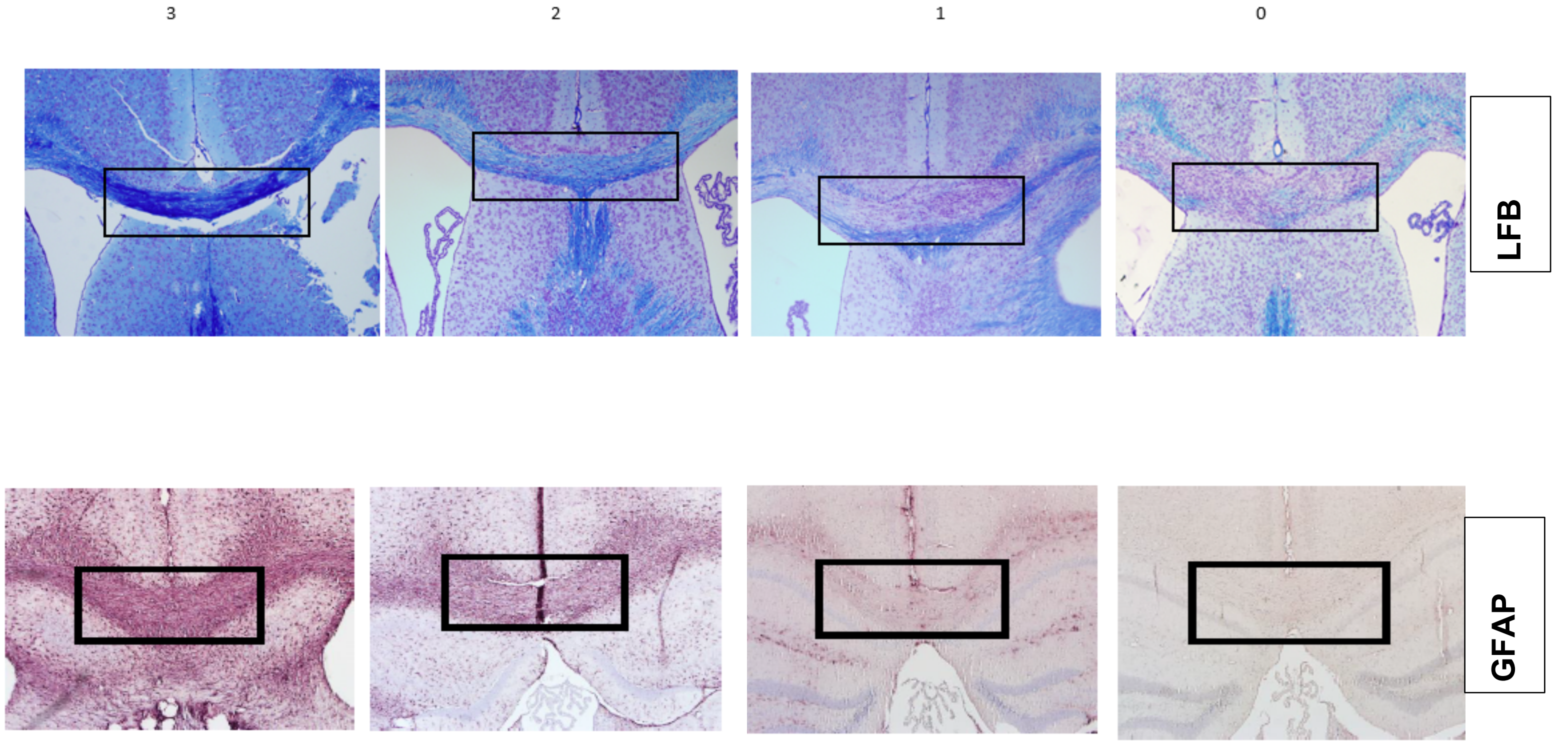

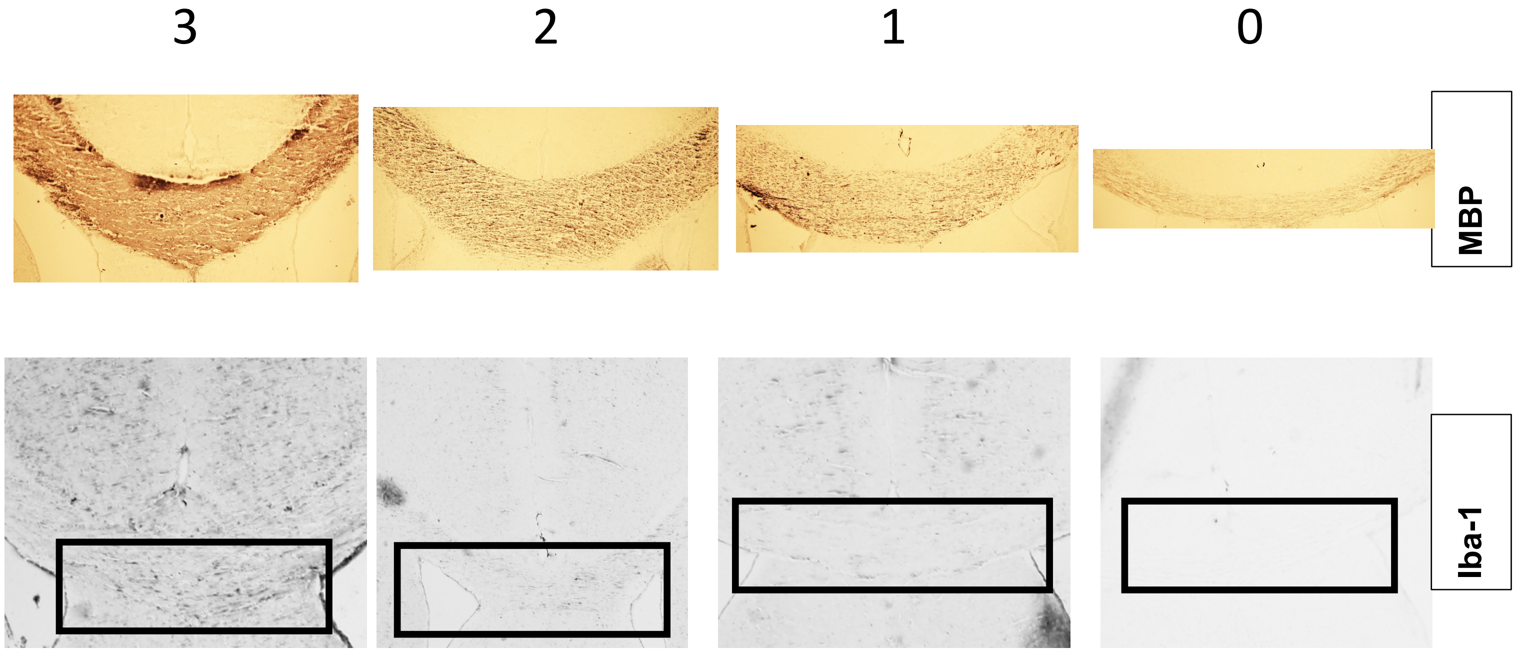
3
2
1
0
MBP
Iba-1
