# Supplementary material for: Clozapine administration enhanced functional recovery after cuprizone demyelination
Source: PLoS One. 2019 May 9;14(5):e0216113. doi: 10.1371/journal.pone.0216113 (PMC6508663; doi:10.1371/journal.pone.0216113)
Supplement: S2 Fig — Clozapine vehicle did not affect weight loss (a), rotarod performance (b), demyelination (c), or astrocyte activation (d) after 6 week treatment + 2 week recovery with clozapine vehicle and cuprizone compared to cuprizone alone. (a) n = 4/group; (b) no treatment (n = 6), cup alone (n = 7), and cup + veh (n = 6); (c) and (d) n = 2 per group, one experiment. (PDF) [file pone.0216113.s002.pdf]

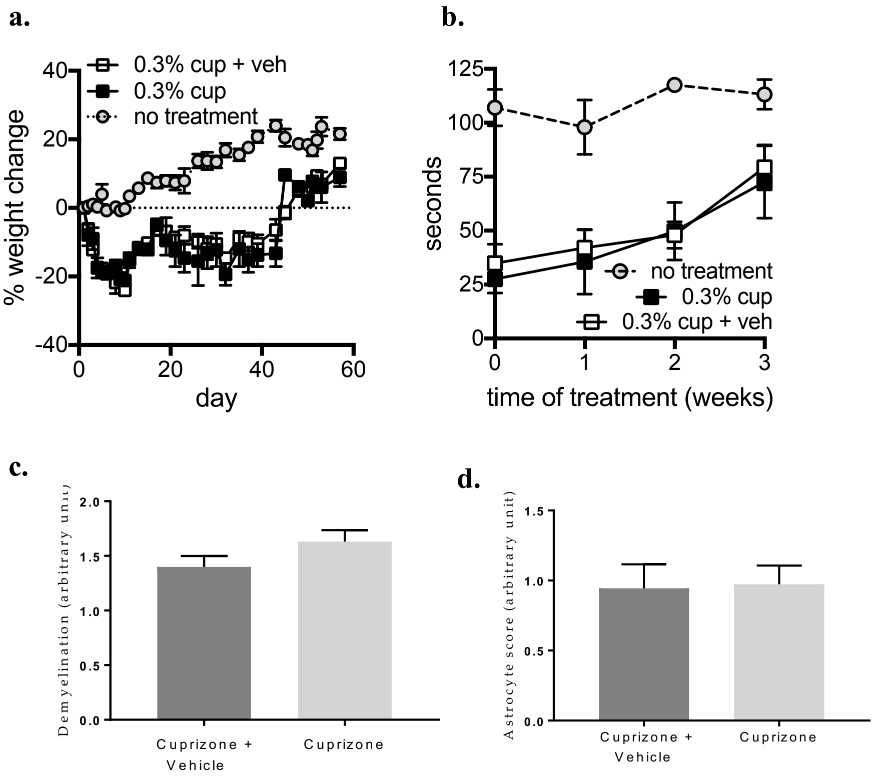

<u>Supplementary Figure 2: Clozapine vehicle did not alter any disease parameters during or after cuprizone intoxication.</u> Clozapine vehicle did not affect weight loss (a), rota-rod performance (b), demyelination (c), or astrocyte activation (d) after 6 week treatment + 2 week recovery with clozapine vehicle and cuprizone compared to cuprizone alone. (a) n = 4/group; (b) no treatment (n = 6), cup alone (n=7), and cup + veh (n = 6); (c) and (d) n = 2 per group, one experiment.
