# Supplementary material for: Clozapine administration enhanced functional recovery after cuprizone demyelination
Source: PLoS One. 2019 May 9;14(5):e0216113. doi: 10.1371/journal.pone.0216113 (PMC6508663; doi:10.1371/journal.pone.0216113)
Supplement: S4 Fig — Clozapine reduced microglial activation (a) and enhanced myelination (b) 1 week after cessation of cuprizone administration. C57Bl/6 mice were fed 0.3% cuprizone diet for 6 weeks or a normal diet. Cuprizone-treated mice were treated with clozapine or vehicle beginning after 5 weeks of cuprizone intoxication. After two weeks of treatment (timepoint noted by ☆ in Fig 3A), mice were euthanized and 5–7 μm sections of corpus callosum were stained for Iba-1 (a) or MBP (b). Shown are representative images used to assess Iba-1 and MBP expression by ImageJ quantitation (Fig 5) or blinded observers (S5 Fig). (PDF) [file pone.0216113.s004.pdf]

S5).

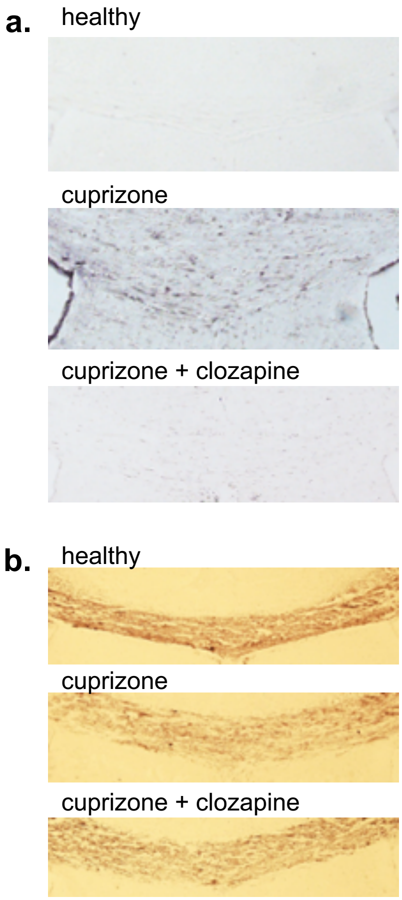

Supplementary Figure 4: Clozapine reduced microglial activation (a) and enhanced myelination (b) 1 week after cessation of cuprizone administration. C57Bl/6 mice were fed 0.3% cuprizone diet for 6 weeks or a normal diet. Cuprizone-treated mice were treated with clozapine or vehicle beginning after 5 weeks of cuprizone intoxication. After two weeks of treatment (timepoint noted by ☆ in Fig 3a), mice were euthanized and 5-7 µm sections of corpus callosum were stained for Iba-1 (**a**) or MBP (**b**). Shown are representative images used to assess Iba-1 and MBP expression by ImageJ quantitation (Fig 5) or blinded observers (Fig S5).
