# Supplementary material for: Clozapine administration enhanced functional recovery after cuprizone demyelination
Source: PLoS One. 2019 May 9;14(5):e0216113. doi: 10.1371/journal.pone.0216113 (PMC6508663; doi:10.1371/journal.pone.0216113)
Supplement: S5 Fig — Sections (★ in Fig 3A) were scored by individuals blinded to their treatment groups using a scale from 0 (low) to 3 (high) for myelin by LFP (a), MBP (b), Iba-1 (c), and GFAP (d). Shown are the means and SEM of individual mice with (a & d) n = 12 for cup and cup + cloz, n = 4 untreated and (b & c) n = 7 for cup, n = 8 cup + cloz, and n = 3 untreated from 2 independent experiments. Kruskal-Wallis test with Dunn’s multiple comparison test (****p<0.000, **p<0.01, and *p<0.05). (PDF) [file pone.0216113.s005.pdf]

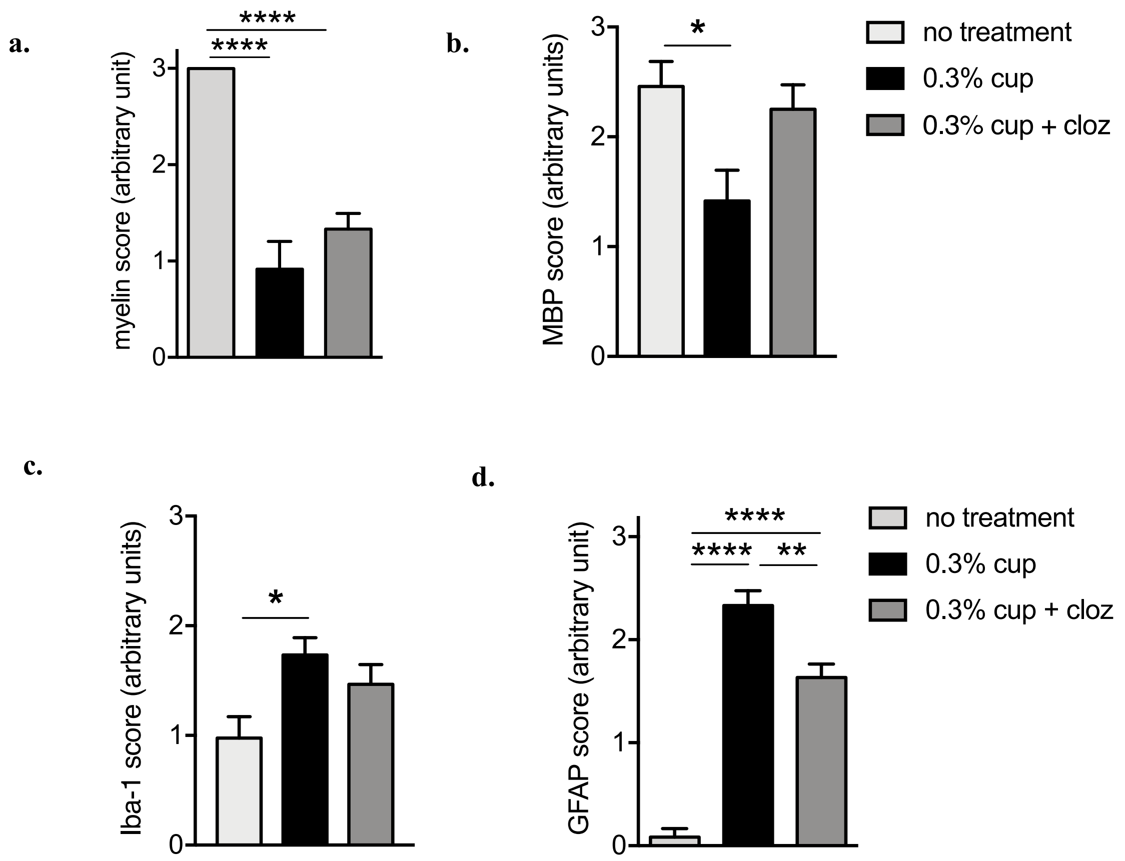

Supplementary Figure 5: Clozapine enhanced myelination and reduced microglial and astrocyte activation 1 week after cessation of cuprizone administration. Sections (timepoint noted by ☆ in Fig 3a) were scored by individuals blinded to their treatment groups using a scale from 0 (low) to 3 (high) for myelin by LFP (a), MBP (b), Iba-1 (c), and GFAP (d). Shown are the means and SEM of individual mice with (a & d) n=12 for cup and cup + cloz, n= 4 untreated and (b & c) n=7 for cup, n=8 cup + cloz, and n= 3 untreated from 2 independent experiments. Kruskal-Wallis test with Dunn's multiple comparison test (****p<0.000, **p<0.01, and *p<0.05).
