# Supplementary material for: Clozapine administration enhanced functional recovery after cuprizone demyelination
Source: PLoS One. 2019 May 9;14(5):e0216113. doi: 10.1371/journal.pone.0216113 (PMC6508663; doi:10.1371/journal.pone.0216113)
Supplement: S6 Fig — C57Bl/6 mice were fed 0.3% cuprizone diet for 6 weeks (+ cuprizone) or a normal diet (Cont.). Cuprizone-treated mice were treated with clozapine (Cloz) or untreated (UT) beginning week 5 of cuprizone intoxication. After two weeks of treatment mice were euthanized and 5–7 μm sections of corpus callosum were stained for mature oligodendrocytes using GST-pi transferase (indicated by arrows in a). The number of oligodendrocytes in the corpus callosum were counted by blinded observers (b). ***p<0.001 One-way ANOVA with Tukey’s post-test compared to all other groups (control n = 5, UT and Cloz-treated n = 4 per group). (PDF) [file pone.0216113.s006.pdf]

a.

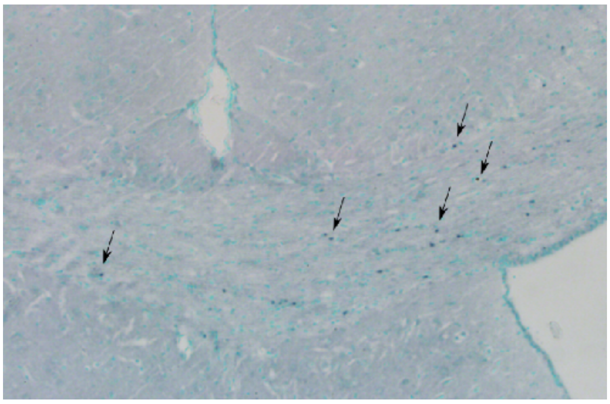

b.

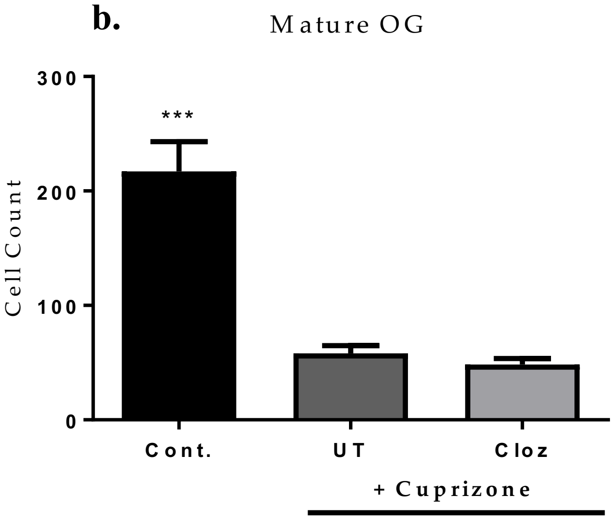

Supplementary Figure 6: Following two weeks of clozapine treatment, clozapine did not significantly affect oligodendrocyte numbers. C57Bl/6 mice were fed 0.3% cuprizone diet for 6 weeks (+ Cuprizone) or a normal diet (Cont.). Cuprizone-treated mice were treated with clozapine (Cloz) or untreated (UT) beginning week 5 of cuprizone intoxication. After two weeks of treatment mice were euthanized and 5-7 μm sections of corpus callosum were stained for mature oligodendrocytes using GST-pi transferase (indicated by arrows in a). The number of oligodendrocytes in the corpus callosum were counted by blinded observers using the ImageJ cell counter plug-in (b). ***$p<0.001$ one-way ANOVA with Tukey's post-test compared to all other groups (control n=5, UT and Cloz treated n=4 per group).
